# Mechanisms and models of TDP-43 proteinopathies

**DOI:** 10.1186/1750-1326-7-S1-L19

**Published:** 2012-02-07

**Authors:** Leonard Petrucelli

**Affiliations:** 1 Mayo Clinic Jacksonville, USA

## Background

Abnormal distribution, modification and aggregation of transactivation response DNA-binding protein 43 (TDP-43) are the hallmarks of multiple neurodegenerative diseases, especially frontotemporal lobar degeneration with ubiquitin-positive inclusions (FTLD-U) and amyotrophic lateral sclerosis (ALS).

## Results

To explore the pathogenic properties of mutant forms of TDP-43, we generated and characterized two mouse lines expressing human TDP-43 carrying the M337V mutation (hTDP-43_M337V_). We found hTDP-43_M337V_ was expressed primarily in the nuclei of neurons in the brain and spinal cord, intranuclear and cytoplasmic phosphorylated TDP-43 aggregates were frequently detected, and the levels of TDP-43 LMW products of ~25 kDa and ~35 kDa species were also increased. Overexpression of hTDP-43_M337V_ dramatically down regulated the levels of mouse TDP-43 (mTDP-43) protein and RNA, indicating TDP-43 levels are tightly controlled in mammalian systems. TDP-43_M337V_ mice displayed reactive gliosis, widespread ubiquitination, chromatolysis, gait abnormalities, and early lethality. Abnormal cytoplasmic mitochondrial aggregates and abnormal phosphorylated tau were also detected in the mice.

## Conclusion

While overexpression of hTDP-43 in wild-type TDP-43 and TDP-43_M337V_ mouse models produces similar phenotypes, our results suggest that overexpression of the hTDP-43_M337V_ can cause neuronal dysfunction due to its effect on a number of cell organelles and proteins, such as mitochondria and TDP-43, that are critical for neuronal activity.

